# Continuous Positive Airway Pressure (CPAP) face-mask ventilation is an easy and cheap option to manage a massive influx of patients presenting acute respiratory failure during the SARS-CoV-2 outbreak: A retrospective cohort study

**DOI:** 10.1371/journal.pone.0240645

**Published:** 2020-10-14

**Authors:** Sophie Alviset, Quentin Riller, Jérôme Aboab, Kelly Dilworth, Pierre-Antoine Billy, Yannis Lombardi, Mathilde Azzi, Luis Ferreira Vargas, Laurent Laine, Mathilde Lermuzeaux, Nathalie Mémain, Daniel Silva, Tona Tchoubou, Daria Ushmorova, Hanane Dabbagh, Simon Escoda, Rémi Lefrançois, Annelyse Nardi, Armand Ngima, Vincent Ioos

**Affiliations:** 1 Service de Médecine Intensive Réanimation, Hôpital Delafontaine, Saint-Denis, France; 2 Service d’Anesthésie, Centre Hospitalier Universitaire de Grenoble, La Tronche, France; 3 Laboratoire de Microbiologie, Hôpital Delafontaine, Saint-Denis, France; 4 Service d’Anesthésie, Hôpital Delafontaine, Saint-Denis, France; 5 Service de Pédiatrie, Hôpital Delafontaine, Saint-Denis, France; 6 Service des Maladies infectieuses, Hôpital Delafontaine, Saint-Denis, France; 7 Service de Pneumologie, Hôpital Delafontaine, Saint-Denis, France; 8 Service des Urgences, Hôpital Delafontaine, Saint-Denis, France; Erasmus Medical Center, NETHERLANDS

## Abstract

**Introduction:**

Because of the COVID-19 pandemic, intensive care units (ICU) can be overwhelmed by the number of hypoxemic patients.

**Material and methods:**

This single centre retrospective observational cohort study took place in a French hospital where the number of patients exceeded the ICU capacity despite an increase from 18 to 32 beds. Because of this, 59 (37%) of the 159 patients requiring ICU care were referred to other hospitals. From 27th March to 23rd April, consecutive patients who had respiratory failure or were unable to maintain an SpO2 > 90%, despite receiving 10–15 l/min of oxygen with a non-rebreather mask, were treated by continuous positive airway pressure (CPAP) unless the ICU physician judged that immediate intubation was indicated. We describe the characteristics, clinical course, and outcomes of these patients. The main outcome under study was CPAP discontinuation.

**Results:**

CPAP was initiated in 49 patients and performed out of ICU in 41 (84%). Median age was 65 years (IQR = 54–71) and 36 (73%) were men. Median respiratory rate before CPAP was 36 (30–40) and median SpO2 was 92% (90–95) under 10 to 15 L/min oxygen flow. Median duration of CPAP was 3 days (IQR = 1–5). Reasons for discontinuation of CPAP were: intubation in 25 (51%), improvement in 16 (33%), poor tolerance in 6 (12%) and death in 2 (4%) patients. A decision not to intubate had been taken for 8 patients, including the 2 who died while on CPAP. Two patients underwent less than one hour CPAP for poor tolerance. In the end, 15 (38%) out of 39 evaluable patients recovered with only CPAP whereas 24 (62%) were intubated.

**Conclusions:**

CPAP is feasible in a non-ICU environment in the context of massive influx of patients. In our cohort up to 1/3 of the patients presenting with acute respiratory failure recovered without intubation.

## Introduction

The pandemic of novel coronavirus disease 2019 (COVID-19) began in Wuhan, China in December 2019. As of August 2th 2020, the WHO reported a total of 17 660 523 COVID-19 cases globally, including 680 894 deaths. In a large UK cohort, death from COVID-19 was strongly associated with being male, older age, deprivation, uncontrolled diabetes and severe asthma [1].

The nature of the pulmonary lesions triggered by SARS-CoV-2 is still a matter of debate. Some histopathological studies suggest that diffuse alveolar damage is not the single pattern [2, 3]. Disorders of the pulmonary circulation (thrombosis, endothelial injury) and organizing pneumonia may also be present. The classical clinical features of ARDS after intubation such as low pulmonary compliance are not found in all patients [4, 5].

In terms of clinical management, initial recommendations suggested early intubation and ARDS-type ventilator settings [6]. Although some studies suggest a role for non-invasive ventilation (NIV) in mild ARDS [7–10], including a recent meta-analysis [11], invasive mechanical ventilation remains the standard of care, especially for severe cases. While CPAP in cardiogenic pulmonary oedema has been shown to reduce intubation rate [12], a randomized trial in acute hypoxemic respiratory failure, showed no effect of CPAP in reducing intubation rate and mortality, despite improved oxygenation [13]. However, during the Chinese and European COVID-19 outbreaks, a number of critical care teams proposed using high flow oxygen through nasal cannula (HFONC) or NIV at least for initial management [14–18]. Optimal respiratory support for COVID-19 patients presenting with acute hypoxemic respiratory failure, however, remains unknown.

**Fig 1:**
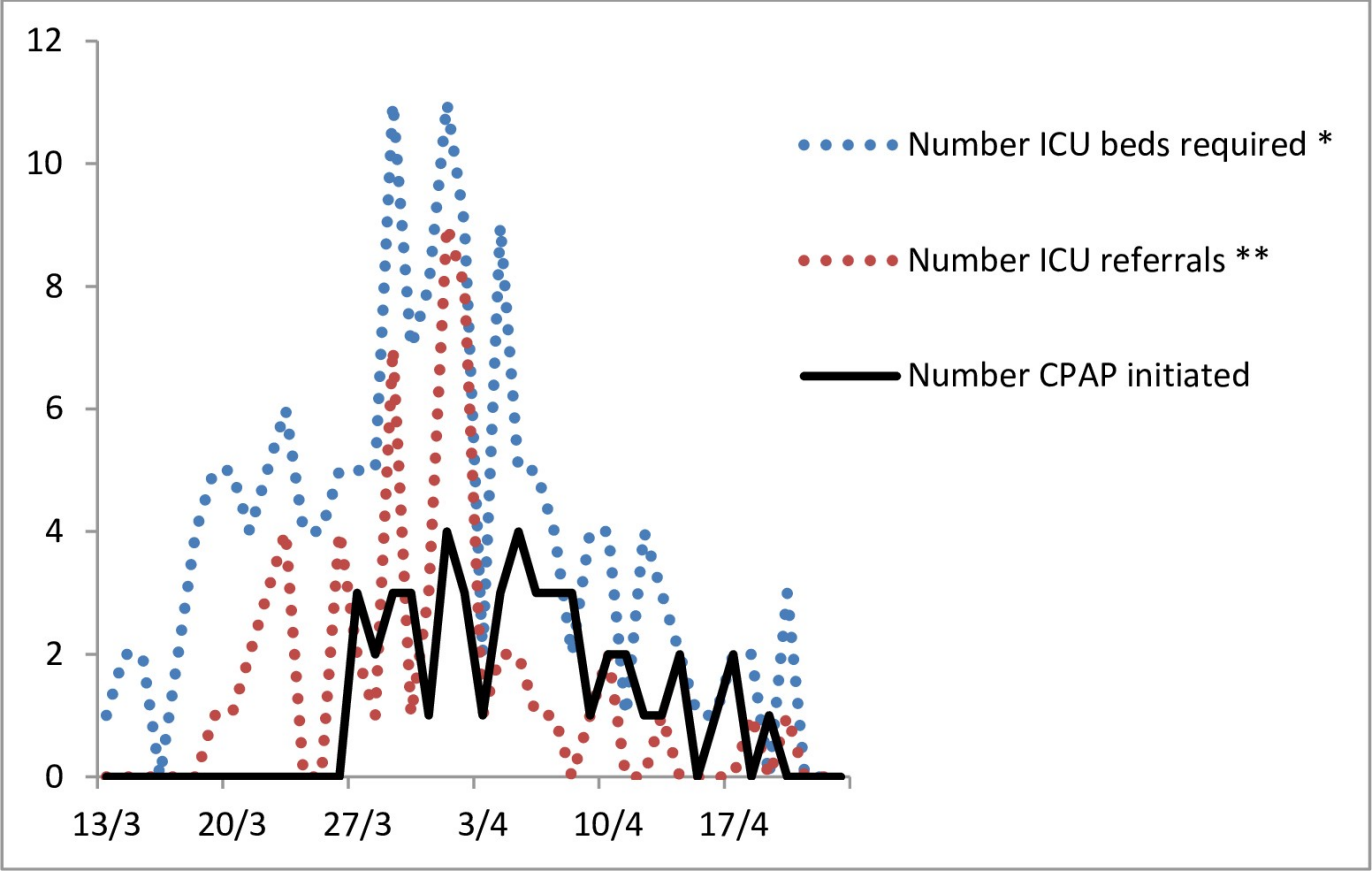
ICU patient load in Delafontaine Hospital during SARS-CoV-2 outbreak. * Total number of patients who were intubated in ED or wards, were admitted in ICU or for whom CPAP was initiated. ** Number of patients that required transfer in other hospitals by emergency medical retrieval service (SAMU).

The district of Seine Saint Denis has been the worst affected area during the 2020 SARS-CoV-2 outbreak in Parisian region, with a mortality in excess of 168.7% as compared to the same period in 2019 [19]. It is densely populated and has a high-deprivation index. From mid-March until end of April 2020, the Delafontaine Hospital, a large public hospital in Saint Denis, experienced a massive influx of patients requiring invasive ventilation. Both intensive care unit (ICU) and Emergency Department (ED) were quickly overwhelmed. The number of patients admitted in the wards (210 non-ICU beds, for 585 COVID-19 admissions) exceeded our ICU capacity (18 beds, increased to 32 during the crisis, for 100 admissions). Fifty-nine (37%) out of the 159 patients requiring ICU care during this period had to be referred to other hospitals (Fig 1). Therefore, we had an urgent need to delay the course of respiratory failure in the less severe patients in order to manage the flow of patients in the ICU and the ED resuscitation room.

To achieve this, we considered face-mask CPAP because it does not require a ventilator. From 27th march onwards, patients with signs of respiratory failure despite 10 to 15 l/min of oxygen delivered by non-rebreather mask (NRM) were systematically assessed for face-mask CPAP or immediate intubation.

In this single centre retrospective observational cohort study, we describe the characteristics and outcomes of patients supported with CPAP in our hospital during the SARS-CoV-2 outbreak.

## Materials and methods

### Study design and participants

We reviewed the characteristics, clinical course and outcomes of all consecutive adults with proven COVID-19 treated with face-mask CPAP in ICU or in wards between 27th March and 23 April. During this 4 weeks-period, patients receiving 10–15 l/min oxygen through NRM who had clinical signs of respiratory failure or were unable to maintain an SpO2 > 90% were treated with face-mask CPAP unless the ICU physician judged that immediate intubation was indicated. Every patient included had a thoracic CT scan suggestive of COVID-19 pneumonia and/or a positive SARS-CoV-2 PCR on naso-pharyngeal swab or broncho-alveolar lavage. The primary outcome under study was reason for CPAP discontinuation (poor tolerance, intubation, death or improvement). Poor tolerance was defined as a refusal by the patient to do more CPAP sessions, because of breathing discomfort.

### Data collection

The following baseline patient characteristics were retrieved from patient electronic medical record: sex, age, comorbidities, body mass index (BMI), withholding / withdrawal of life-sustaining therapies, associated COVID-19 therapies administered before the primary outcome under study occurred (antivirals, corticosteroids, immuno-modulating therapies, prone positioning), oxygen flow rate and SpO2 before and after starting CPAP treatment, duration of CPAP treatment, medical unit where CPAP treatment was performed, duration of invasive mechanical ventilation, SAPS2 score for patients admitted in ICU, driving pressure and P/F ratio on first day of mechanical ventilation. The clinical outcomes (i.e. discharges from hospital, mortality) were recorded until the final day of follow-up on June 24^th^.

### CPAP therapy

CPAP of 5 to 10 cm H2O was delivered via a face-mask dedicated to NIV (Performa Track®) with one of 2 types of CPAP valve (Boussignac™ or CPAP-O-two™) or alternatively, an ICU ventilator (Servo I® or Evita Infinity V500®). Treatment was undertaken in a medical ward, the ED short-stay unit or the ICU. An electrostatic heat and moisture exchanger filter (DAR™) was placed between the mask and the CPAP valve to prevent aerosolization of virus through expired gases. All patients were admitted to a single room with implementation of contact and airborne precautions. Medical and nursing staff in wards, unfamiliar with NIV, were trained by the intensivist who was initiating the CPAP treatment. Patients received an initial prolonged session lasting at least 4 hours before being reassessed of their need of invasive mechanical ventilation. If the patient could be temporarily taken off CPAP without an immediate fall of SpO2 below 90% (on O2 15l/min via NRM) or recurrence of clinical signs of acute respiratory failure, CPAP treatment was resumed for 2 hours every 4 hours. Progressive weaning of CPAP was performed according to clinical signs, pulse oximetry and arterial blood gases. When possible, patients were managed in the ICU (nurse/patient ratio 1:2). If no ICU bed was available (as in over 80% cases), patients with CPAP were shifted to the ED short-stay unit (8 beds) adjacent to the ICU (nurse/patient ratio 1:4) which allowed frequent re-evaluation of the patient’s state by the intensivist on duty. In the eventuality of no available bed in the ED short stay unit, CPAP treatment was instituted and managed in the medical ward were the patient had been admitted (nurse/patient ratio 1:7 during the outbreak). Ward patients on CPAP were systematically reviewed overnight by the resident on duty responsible for the COVID-19 medical wards.

### Statistics

No *a priori* statistical sample size calculation was performed. Sample size was equal to the number of patients treated during the study period. Quantitative values are expressed as the median (interquartile range, IQR), and qualitative values are presented as numbers (percentages). Univariate analysis was performed using Fisher exact test or Wilcoxon test, as appropriate. All tests were two-sided and a p value <0.05 was considered statistically significant. Because of alpha inflation due to multiple comparisons, findings should be interpreted as exploratory. A Cox hazard proportional model was fit for time to intubation, controlling for potential confounders in the cohort of 39 patients analysed. All variables available at baseline and associated with intubation in univariate analysis with a p-value <0.10 were selected. Variables selected are: CT-scan severity (<50% vs ≥50% of lung involved), SpO_2_ at the time of CPAP initiation, dose of anticoagulant (simple, double or curative) and time between hospital admission and CPAP initiation. Because of the important differences in the proportion of patients on corticosteroids in the 2 groups (though statistically non-significant in univariate analysis) and the impact on mortality of corticosteroid treatment found in the Recovery trial [20], we included it as an additional variable in the model. Variables with more than 10% missing values were not implemented in the multivariate analysis. The analyses were carried out using R version 3.6.2 (The R Project For Statistical Computing, Vienna, Austria; http://www.R-project.org).

### Ethics

The study was approved by the national ethics review board (CNRIPH—Commission Nationale des Recherches Impliquant la Personne Humaine) under the number 2020-A01396-33. The ethic committee waived the requirement for informed consent: patients or their next-of-kin were informed by mail about the data collection process and their right to oppose. The database was declared to the Commission Nationale Informatique et Libertés (CNIL) under the number 2217928. Electronic medical records of the Delafontaine hospital (Saint-Denis, France), concerning patients who sought care between March and April 2020, were accessed between May and June 2020. Statistical analyses were conducted on anonymised data.

## Results

**Fig 2:**
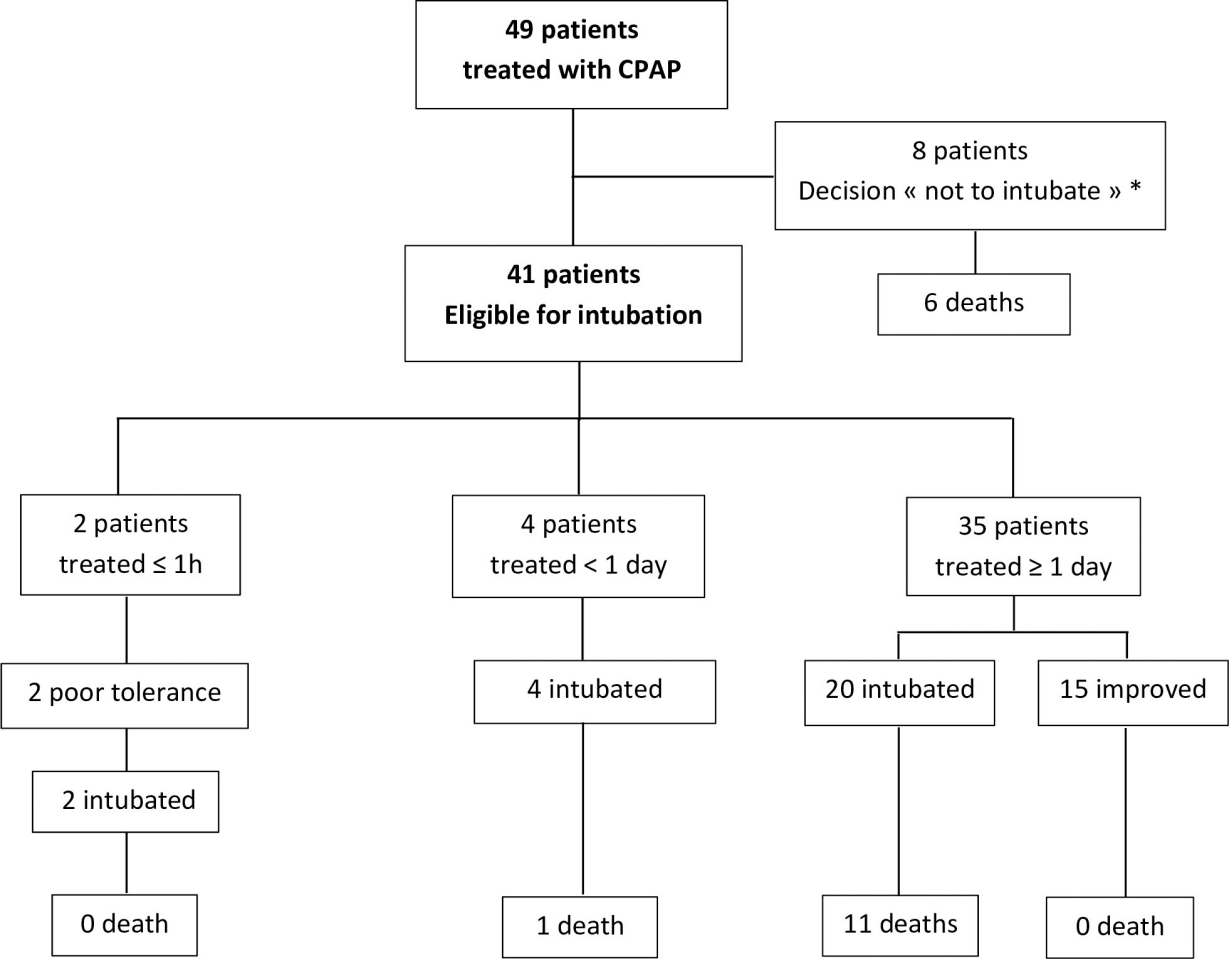
CPAP therapy—patient flow diagram. * CPAP discontinued for poor tolerance (5 patients), death during treatment (2 patients) and improvement (1 patient).

Forty-nine consecutive patients were treated with CPAP between 27^th^ March and 23^rd^ April 2020 (Fig 2). Initiation of CPAP occurred throughout the entire study period and followed the epidemic curve (Fig 1). SARS-CoV-2 pneumonia was confirmed by PCR in 39 (79%) patients and by thoracic CT scan in all patients. Twenty-six (53%) patients were eventually intubated and a total of 18 (37%) died.

Patients’ characteristics are presented in Table 1. The median age was 65 years (IQR = 54–71) and 36 (73%) were men. Forty-one (84%) patients had at least one comorbidity. The most frequent were hypertension (31 patients, 63%), obesity (13 patients, 34%) and diabetes (16 patients, 33%). The median duration of symptoms before hospital admission was 6 days (IQR = 5–9). Thoracic CT-scan at admission showed mild (10 to 25%), moderate (25 to 50%) or severe (>50%) lung involvement in 13 (27%), 23 (46%) and 13 (27%) patients respectively.

**Table 1:** Patients’ characteristics.

Characteristics	All patients (n = 49)	Patients improved with CPAP (n = 15) *	Patients intubated after CPAP (n = 24) *	*P* value ^δ^
Age in year ^ε^	65 (54–71)	67 (53–68.5)	62(54–69)	.79
Age categories ^η^				
0–39 yr	0	0	0	
yr	5/49 (10%)	1/15 (7%)	3/24 (12.5%)	1
50–59 yr	11/49 (22%)	4/15 (27%)	6/24 (25%)	1
60–69 yr	19/49 (39%)	8/15 (53%)	9/24 (37.5%)	.51
70–79 yr	10/49 (20%)	2/15 (13%)	5/24 (20%)	.69
≥80 yr	4/49 (8%)	0	1/24 (5%)	1
Female sex ^η^	13/49 (27%)	4/15 (27%)	5/24 (20%)	.71
BMI distribution ^η^				
< 24,9 kg/m^2^	10/38 (26%)	3/11 (27%)	6/21 (29%)	1
25–29,9 kg/m^2^	15/38 (40%)	3/11 (27%)	9/21 (43%)	.46
30–34,9 kg/m^2^	8/38 (21%)	3/11 (27%)	4/21 (20%)	.67
35–39,9 kg/m^2^	2/38 (5%)	0	1/21 (4%)	1
≥40 kg/m^2^	3/38 (8%)	2/11 (19%)	1/21 (4%)	.27
Comorbidities ^η^				
Any	41/49 (84%)	11/15 (73%)	20/24 (83%)	.69
Hypertension	31/49 (63%)	9/15 (60%)	16/24 (67%)	.74
Diabetes	16/49 (33%)	6/15 (40%)	7/24 (29%)	.51
Cerebrovascular disease	3/49 (6%)	1/15 (7%)	2/24 (8%)	1
Coronary artery disease	2/49 (4%)	0	0	
Chronic renal failure	5/49 (10%)	2/15 (13%)	3/24 (12.5%)	1
Chronic lung disease	10 (20%)	2/15 (13%)	4/24 (17%)	1
Cancer	1 (2%)	0	0	
Immunodeficiency	0	0	0	
Delay between symptoms and hospital admission (days) ^ε^	6 (5–9)	6 (5.5–9.5)	6 (5–8.25)	.76
SARS-CoV-2 PCR positivity ^η^	39/47 (83%)	10/14 (71%)	21/24 (88%)	.39
Thoracic CT at admission ^η^				
10–25%	13/49 (27%)	7/15 (47%)	4/24 (17%)	.07
25–50%	23/49 (46%)	6/15 (40%)	13/24 (54%)	.75
>50%	13/49 (27%)	2/15 (13%)	7/24 (29%)	.15

* Patients excluded for analysis (n = 10): withdrawal/limitations of life-sustaining therapies (n = 8), <1 hour CPAP treatment (n = 2).

^ε^ Median (IQR).

^η^ Number / total number (%).

Modalities of CPAP therapy and associated interventions are described in Table 2. CPAP was performed out of ICU in 41 (84%) cases. Median duration of CPAP therapy was 3 days (IQR = 1–5). Reasons for discontinuation of CPAP were intubation for invasive mechanical ventilation in 25 (51%) patients, improvement in 16 (33%), poor tolerance in 6 (12%) and death in 2 (4%). A decision not to intubate had been taken with the patient and their family for the 2 patients who died while on CPAP. All patients received at least once daily prophylactic anticoagulation. Twice daily (thus double dose) prophylactic anticoagulation, typically enoxaparin 40mg every 12 hours, was administered in 19 (39%) patients while 14 (29%) received therapeutic anticoagulation. Hydroxychloroquine was administered in 17 (35%) patients, Lopinavir/Ritonavir in 4 (8%), corticosteroids in 29 (59%) and Anakinra in 7 (14%). Awake prone positioning was used in 7 (14%) patients. Two of those were eventually intubated.

**Table 2:** CPAP therapy and other interventions (before or during CPAP period).

	All patients	Patients improved with CPAP (n = 15)*	Patients intubated after CPAP (n = 24)*	*P* value
Time between admission and CPAP initiation in days ^ε^	3 (1–5)	4 (4–6.5)	2 (1–5)	**.04**
Care zone of initiation of CPAP				
ICU	8/49 (16%)	2/15 (13%)	5/24 (21%)	.69
ED short stay unit	29/49 (59%)	9/15 (61%)	16/24 (67%)	.74
COVID-19 ward	12/49 (25%)	4/15 (26%)	3/24 (12%)	.69
CPAP device				
Boussignac™ valve	41/49 (84%)	11/15 (74%)	20/24 (83%)	.69
CPAP-O-two™ valve	3/49 (6%)	2/15 (13%)	1/24 (4%)	.55
ICU ventilator	5/49 (10%)	2/15 (13%)	3/24 (13%)	1
Oxygen flow rate before CPAP initiation				
15L/min	42/47 (89%)	11/15 (73%)	22/22 (100%)	.05
11-12L/min	5/47 (11%)	4/15 (27%)	0	.05
SpO2 in % before CPAP initiation ^ε^	92 (90–95) n = 44	95 (92.5–95.5) n = 15	92 (90–93) n = 21	**.02**
Respiratory rate per min before CPAP ^ε^	36 (30–40) n = 36	38 (29–40) n = 13	32 (30–38) n = 19	.47
Oxygen flow on CPAP in L/min	25 (23–25) n = 21	25 (23–26) n = 5	25 (25–26) n = 12	1
SpO2 in % on CPAP ^ε^	97 (94–98) n = 29	98 (96–98) n = 9	96 (93–98) n = 15	.14
Respiratory rate per min on CPAP^ε^	34 (29–37) n = 23	29 (23–32) n = 6	36 (30–37) n = 14	.46
CPAP duration in days ^ε^	3 (1–5)	4 (3–7)	2 (2–3)	**.002**
<1h ^η^	4/49 (8%)	-	-	
1h - 1day ^η^	6/49 (12%)	0	4/24 (17%)	.15
1–5 days ^η^	30/49 (61%)	10/15 (67%)	19/24 (79%)	.46
>5 days ^η^	9/49 (18%)	5/15 (33%)	1/24 (4%)	**.02**
Other interventions				
Antibiotics°	45/49 (92%)	13/15 (87%)	23/24 (96%)	.55
Lopinavir/ritonavir	4/49 (8%)	1/15 (7%)	1/24 (4%)	1
Corticosteroids	29/49 (59%)	10/15 (67%)	11/24 (46%)	.32
Anakinra	7/49 (14%)	2/15 (13%)	3/24 (13%)	1
Hydroxychloroquine	17/49 (35%)	6/15 (40%)	9/24 (38%)	1
Therapeutic anticoagulation	14/49 (29%)	3/15 (20%)	7/24 (29%)	.71
Twice daily (double dose) prophylactic anticoagulation	19/49 (39%)	9/15 (60%)	6/24 (25%)	**.04**
Once daily (single dose) prophylactic anticoagulation	16/49 (32%)	3/15 (20%)	11/24 (46%)	.17

* Patients excluded for analysis (n = 10): withdrawal/limitations of life-sustaining therapies (n = 8), <1 hour CPAP treatment (n = 2).

^ε^ Median (IQR).

^η^ Number / total number (%).

° Antibiotics: Amoxicillin-clavulanic acid, Spiramycin, Azithromycin, Erythromycin, third generation cephalosporin, Piperacillin-tazobactam, Cefepim.

Eight patients had a withdrawal or limitation of life-sustaining therapies (“do-not intubate” decision). Of the 41 other patients, 2 had poor tolerance of CPAP, resulting in its discontinuation within less than one hour. We did not consider these patients as being significantly treated, which left 39 patients suitable for analysis of outcome. Fifteen patients (38%) out of 39 showed sustained clinical improvement with CPAP therapy and never required intubation, including 1 patient (25%) in the 40–49 years age category, 4 patients (40%) in the 50–59 years, 8 patients (47%) in the 60–69 years and 2 patients (29%) in the 70–79 years. The other 24 patients (62%) eventually required invasive ventilation (Fig 2). In this group, median time from CPAP initiation to intubation was 1 day (IQR = 1–2), median P/F immediately after intubation was 100 (IQR = 80–139), and median duration of mechanical ventilation was 17 days (IQR = 10–22).

**Fig 3:**
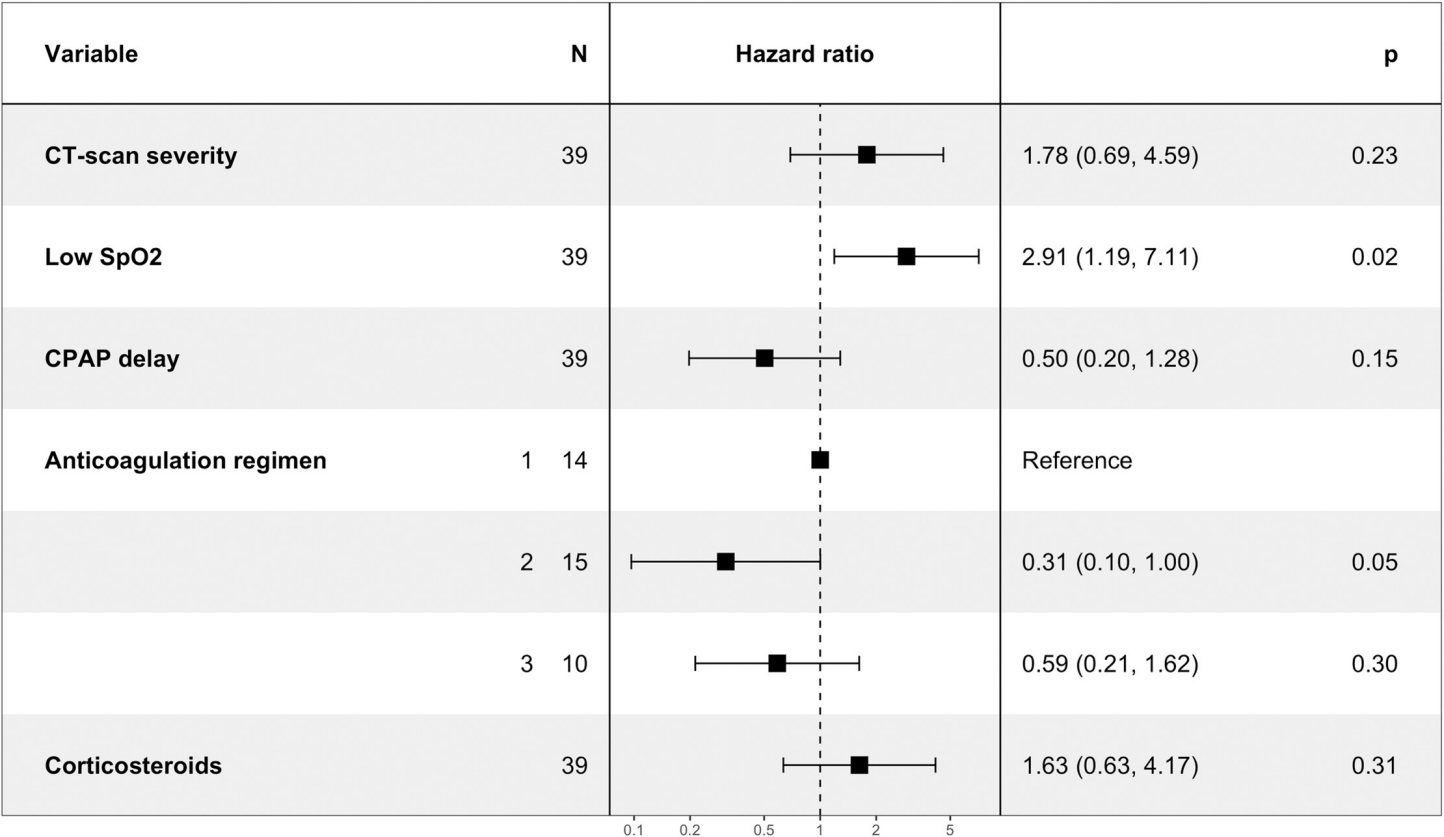
Factors associated with intubation. Hazard ratio of intubation adjusted for CT-scan severity (more or less of 50% of lung involved by SARS—CoV2 induced lesions), low saturation (SpO2, < 92% or > 92%), delay in days between hospitalization and CPAP initiation (two groups based on the median value of CPAP delay), use of anticoagulant treatment grouped by dosage: simple dose prophylaxis (1), double dose prophylaxis (2) or curative treatment (3) and treatment with corticosteroids. *P* values are indicated as the result of likelihood-ratio test. The validity of the proportional hazards assumption was tested using cox.zph() function in R (P values > 0.05) and by visualization of Schoenfeld residuals.

Patients who improved with CPAP were compared to patients who required invasive mechanical ventilation. Characteristics regarding age, sex, comorbidities and disease presentation were similar in both groups. Patient who improved with CPAP were treated later in their hospital stay, had higher oxygen saturation before CPAP initiation, longer duration of CPAP and received more often concomitant double dose prophylactic anticoagulation. In multivariate analysis, only low oxygen saturation before initiation of CPAP was independently associated with a higher risk of intubation (Fig 3).

Twelve (46%) of the 26 intubated patients had a fatal outcome. Median SAPS 2 score of ventilated patients was 57 (IQR = 38–64), resulting in a standardized mortality ratio of 0.75. At the time of final follow up, 18/49 (37%) patients were dead, 30 (61%) were discharged (14 from the group of patients who improved with CPAP), 1 (2%) was still hospitalized in intensive care unit but weaned from mechanical ventilation.

## Discussion

Our hospital experienced a massive influx of hypoxemic patients during the COVID-19 outbreak, 59 (37%) of the 159 patients requiring ICU care had to be referred to other hospitals for lack of ICU beds. In this context we tried CPAP as a temporizing treatment in the management of acute respiratory failure. We choose not to use bi-level pressure NIV for several reasons. First, the number of ventilators available could not ensure surge capacity in the context of massive patients influx, and the CPAP devices were cheap enough to be bought in a large amount (28€ for a Boussignac™, 62€ for a CPAP-O-two™). Second, bi-level pressure modes could have exposed patients who already have increased respiratory drive to the risk of ventilation induced lung injury through excessive tidal volume [21]. Third, the increase in positive pressure during inspiration was suspected to carry a greater risk of aerosolization of virus particles, hence increasing the risk of contamination of health care workers [22, 23]. The final reason was to keep pressure support ventilation as an option for pre-oxygenation before intubation when indicated [24]. Using bi-level pressure modes would have also required more intensive training of ward staff unfamiliar with NIV techniques. HFONC was also not considered because of the lack of high flow oxygen delivery devices during the outbreak period. In addition, there was also a concern about a greater risk of aerosolization, especially in non ICU settings were strict compliance with airborne precautions was more difficult to achieve.

In this single center retrospective observational study, overall mortality of this cohort was 37% (18/49). Sixteen (33%) patients improved with face-mask CPAP, and eventually did not require invasive ventilation though they were very hypoxemic (11 (73%) of them required 15L/min oxygen). Apart from 2 patients with a do-not-intubate orders, no death occurred during CPAP therapy. Mortality rate was 46% in the patient group requiring invasive mechanical ventilation, which was consistent with a large cohort of 20133 patients from 208 hospitals in UK (International Severe Acute Respiratory and emerging Infections Consortium—ISARIC). Among 3001 admitted to critical care (high dependency unit or intensive care unit), mortality was 32% while 41% continued to receive care at the date of reporting. In the 1658 patients receiving invasive ventilation mortality was 37% while 46% were still in hospital [25]. This mortality rate was related to the severity of illness (median SAPS2 score of 57) but may also be due to delayed intubation.

Reports are emerging on the use of CPAP in situations similar to ours during the SARS-Cov-2 pandemic. In a single center in UK, CPAP was initiated in patients requiring more than 28% FiO2 or 4l/mn oxygen in combination with awake proning [26]. When compared to the ISARIC cohort [25], there were reduced ICU admissions (7.2 versus 16.5%) and invasive ventilation rates (4.8 versus 9.8%), with comparable hospital mortality (33.3 versus 36.8%). Another single center retrospective study in UK suggest a positive effect of CPAP therapy, with a favourable outcome (i.e. survival without mechanical ventilation) in 14 (58%) patients, and an intubation rate of 38%, which was similar to our results [27]. A French two period retrospective study favours CPAP over oxygen: intubation-free survival was 77% (29/38) with CPAP compared to 43% (6/14) with oxygen (p = 0,043). However it was performed in the absence of shortage of ICU beds, and CPAP was initiated in patients less hypoxemic than our population (oxygen flow > 6 L/min for Sp02 > 92%), those two points may be the reasons of a lower intubation rate compared to ours [28]. In an Italian prospective cohort, 157 patients underwent helmet CPAP with a 55% survival without mechanical ventilation. In this study, patients were less hypoxemic than in our cohort at CPAP initiation (PaO2/FiO2 < 300 mmHg). The helmet interface was well tolerated with discontinuation in only 4 patients [29].

On the whole, despite the fact that non-invasive ventilation techniques (HFONC, bi-level pressure ventilation or CPAP) have already been used in several respiratory virus outbreaks (SARS, MERS, H1N1), we lack strong evidence on their efficiency because studies were mainly retrospective, with inappropriate control for selecting or confounding bias, or without any control group [30, 31]. As a consequence, recommendations from scientific societies concerning non-invasive oxygen therapy in COVID-19 are heterogenous, some favouring HFONC, CPAP, or bi-level pressure, depending on the country [32].

It is not possible to infer from our study any definite conclusion on the role of CPAP to avoid invasive ventilation because of the small sample size and because we were unable to identify a population of patients that could have been a comparator. Our study has several other limitations. First, due to its retrospective design, we were unable to collect additional data that could have contributed to a better understanding of the role of CPAP in managing hypoxemic respiratory failure in COVID-19. Data on actual pressure levels delivered to each patient and the number of hours per day of CPAP could not be collected. It was also not possible to ascertain in all patients whether vital signs (SpO2, respiratory rate) and arterial blood gases were taken while on CPAP or while on NRM, hence a high rate of missing values. Patients with profound hypoxemia and high respiratory rate on CPAP may be exposed to self-induced lung injury. We attempted to collect the values for driving pressures immediately after intubation and positive expiratory pressure levels during CPAP therapy but these data were unfortunately only available in a few cases. This should be investigated in further studies.

Secondly, due to small sample size, the observed effect of CPAP in avoiding invasive mechanical ventilation within a sub-group of patients could be biased by concomitant treatments (drugs and/or prone positioning during spontaneous breathing) administered to spontaneously breathing-patients. However, in the multivariate analysis, corticosteroid treatment, the main therapy that has been shown to impact mortality [20], was not associated with the success of CPAP treatment. The absence of a control group does not allow us to make any firm conclusion on the role of CPAP in avoiding intubation. In addition, some patients treated with CPAP may simply have received higher FiO2 because the seal of the mask is better and the O2 flow higher as compared to patients on NRM: for example with a Boussignac™ CPAP, O2 flow was usually set between 20 to 30 L/mn to reach the target pressure of 5 to 10 cm H2O. This could have contributed to their clinical improvement.

Third, there might be several selections bias. We chose to include all patients with findings highly suggestive of COVID-19 on thoracic CT scan among which only 79% had a positive PCR on respiratory samples. However, SARS Cov2 rt PCR on naso pharyngeal swabs is known to have an imperfect sensibility, and we considered that at the peak of the outbreak, alternative diagnosis were improbable [33]. The group of patients who did not need mechanical ventilation may have been less severe. This may be an important bias for their favourable outcome, which may not be due to the effect of CPAP only. This is suggested by the higher levels of SpO2 at initiation of CPAP in the group of patients who improved compared with the group of patients who progressed to intubation. Response to CPAP could be used to identify patients who do not require intubation despite being profoundly hypoxemic [34]. However, CPAP could also have worsened the condition of patients whose intubation was delayed. The high SAPS2 scores of the intubated patients in the study provide some evidence to this effect.

Fourth and finally, contamination of health care workers was not evaluated. Expired gases dispersion during CPAP seems to be limited if there is good mask interface fitting [35], but leaks do occur incidentally and NIV is considered an aerosol-generating procedure [23]. Potential benefit from face-mask CPAP should be weighed against the risk of contamination of health care workers, especially in settings were infection prevention and control precautions are difficult to maintain. Choosing the appropriate interface is critical to decrease leaks and minimize aerosolization and there may be some advantages to select full face masks [22, 36]. Helmet is another option but is more difficult to handle in a non-ICU setting [37].

The role of face-mask CPAP in managing acute hypoxemic respiratory failure in COVID-19 patients warrants further investigation in larger prospective studies and comparison with other ways to manage hypoxemic respiratory failure, such as high flow nasal oxygen cannula [14, 16, 17]. The simplicity and practicality of CPAP in a number of contexts, including massive patient influx and resource limited settings, is appealing [38]. However, the likely increased risk of contamination of health care workers, notably if personal protective equipment is inadequate, must be taken in account. CPAP could also be considered as a first-line respiratory support strategy in less hypoxemic patients without significant respiratory failure in association with other strategies to improve oxygenation, such as awake prone positioning [17, 26, 39–44].

## Conclusion

We found that treatment with face-mask CPAP was feasible in a non-ICU environment and in the context of a massive influx of patients. In our situation, it was useful to post-pone intubation and to manage the flow of patient requiring invasive ventilation. We also found, that among patients who have low SpO2 and /or signs of respiratory failure while on 15l/min O2 via NRM more than one third eventually did not need invasive mechanical ventilation. Given the limitations of our study, the role of face-mask CPAP in managing patients with hypoxemic respiratory failure should be investigated in further research.

## Supporting information

S1 Dataset (XLSX)

## References

[1] Collaborative TO, Williamson E, Walker AJ, Bhaskaran KJ, Bacon S, Bates C, et al OpenSAFELY: factors associated with COVID-19-related hospital death in the linked electronic health records of 17 million adult NHS patients. MedRxiv 2020:2020.05.06.20092999. 10.1101/2020.05.06.20092999.

[2] Leisman DE, Deutschman CS, Legrand M. Facing COVID-19 in the ICU: vascular dysfunction, thrombosis, and dysregulated inflammation. Intensive Care Med 2020:1–4. 10.1007/s00134-020-06059-6. PMC7186535 32347323

[3] Copin MC, Parmentier E, Duburcq T, Poissy J, Mathieu D, Caplan M, et al Time to consider histologic pattern of lung injury to treat critically ill patients with COVID-19 infection. Intensive Care Med 2020:1–3. 10.1007/s00134-020-06057-8. PMC7178098 32328726

[4] Gattinoni L, Chiumello D, Caironi P, Busana M, Romitti F, Brazzi L, et al COVID-19 pneumonia: different respiratory treatments for different phenotypes? Intensive Care Med 2020 10.1007/s00134-020-06033-2. PMC7154064 32291463

[5] Marini JJ, Gattinoni L. Management of COVID-19 Respiratory Distress. JAMA 2020 10.1001/jama.2020.6825. 32329799

[6] Alhazzani W, Møller MH, Arabi YM, Loeb M, Gong MN, Fan E, et al Surviving Sepsis Campaign: guidelines on the management of critically ill adults with Coronavirus Disease 2019 (COVID-19). Intensive Care Med 2020;46:854–87. 10.1007/s00134-020-06022-5 32222812 PMC7101866

[7] Antonelli M, Conti G, Moro M, Esquinas A, Gonzalez-Diaz G, Confalonieri M, et al Predictors of failure of noninvasive positive pressure ventilation in patients with acute hypoxemic respiratory failure: a multi-center study. Intensive Care Med 2001;27:1718–28. 10.1007/s00134-001-1114-4 11810114

[8] Antonelli M, Conti G, Esquinas A, Montini L, Maggiore SM, Bello G, et al A multiple-center survey on the use in clinical practice of noninvasive ventilation as a first-line intervention for acute respiratory distress syndrome*. Crit Care Med 2007;35:18–25. 10.1097/01.CCM.0000251821.44259.F3 17133177

[9] Agarwal R, Reddy C, Aggarwal AN, Gupta D. Is there a role for noninvasive ventilation in acute respiratory distress syndrome? A meta-analysis. Respir Med 2006;100:2235–8. 10.1016/j.rmed.2006.03.018 16678394

[10] Luo J, Wang M, Zhu H, Liang B, Liu D, Peng X, et al Can non-invasive positive pressure ventilation prevent endotracheal intubation in acute lung injury/acute respiratory distress syndrome? A meta-analysis. Respirology 2014;19:1149–57. 10.1111/resp.12383 25208731

[11] Ferreyro BL, Angriman F, Munshi L, Del Sorbo L, Ferguson ND, Rochwerg B, et al Association of Noninvasive Oxygenation Strategies With All-Cause Mortality in Adults With Acute Hypoxemic Respiratory Failure. JAMA 2020. 10.1001/jama.2020.9524. PMC7273316 32496521

[12] Bersten AD, Holt AW, Vedig AE, Skowronski GA, Baggoley CJ. Treatment of Severe Cardiogenic Pulmonary Edema with Continuous Positive Airway Pressure Delivered by Face Mask. N Engl J Med 1991;325:1825–30. 10.1056/NEJM199112263252601 1961221

[13] Delclaux C, L’Her E, Alberti C, Mancebo J, Abroug F, Conti G, et al Treatment of Acute Hypoxemic Nonhypercapnic Respiratory Insufficiency With Continuous Positive Airway Pressure Delivered by a Face Mask. JAMA 2000;284:2352 10.1001/jama.284.18.2352 11066186

[14] Geng S, Mei Q, Zhu C, Yang T, Yang Y, Fang X, et al High flow nasal cannula is a good treatment option for COVID-19. Hear Lung 2020 10.1016/j.hrtlng.2020.03.018. PMC7151489 32295710

[15] Ding L, Wang L, Ma W, He H. Efficacy and safety of early prone positioning combined with HFNC or NIV in moderate to severe ARDS: a multi-center prospective cohort study. Crit Care 2020;24:28 10.1186/s13054-020-2738-5 32000806 PMC6993481

[16] Wang K, Zhao W, Li J, Shu W, Duan J. The experience of high-flow nasal cannula in hospitalized patients with 2019 novel coronavirus-infected pneumonia in two hospitals of Chongqing, China. Ann Intensive Care 2020;10:37 10.1186/s13613-020-00653-z 32232685 PMC7104710

[17] Xu Q, Wang T, Qin X, Jie Y, Zha L, Lu W. Early awake prone position combined with high-flow nasal oxygen therapy in severe COVID-19: a case series. Crit Care 2020;24:250 10.1186/s13054-020-02991-7 32448330 PMC7246000

[18] Duca A, Memaj I, Zanardi F, Preti C, Alesi A, Della Bella L, et al Severity of respiratory failure and outcome of patients needing a ventilatory support in the Emergency Department during Italian novel coronavirus SARS-CoV2 outbreak: Preliminary data on the role of Helmet CPAP and Non-Invasive Positive Pressure Ventilati. EClinicalMedicine 2020;24:100419 10.1016/j.eclinm.2020.100419 32766538 PMC7301102

[19] Observatoire Régional de Santé. La surmortalité durant l’épidémie de Covid-19 dans les départements franciliens n.d. https://www.ors-idf.org/nos-travaux/publications/la-surmortalite-durant-lepidemie-de-covid-19-dans-les-departements-franciliens.html (accessed May 18, 2020).

[20] Dexamethasone in Hospitalized Patients with Covid-19—Preliminary Report. N Engl J Med 2020:1–11. 10.1056/nejmoa2021436. PMC7383595 32678530

[21] Brochard L, Slutsky A, Pesenti A. Mechanical ventilation to minimize progression of lung injury in acute respiratory failure. Am J Respir Crit Care Med 2017;195:438–42. 10.1164/rccm.201605-1081CP 27626833

[22] Hui DS, Chow BK, Lo T, Tsang OTY, Ko FW, Ng SS, et al Exhaled air dispersion during high-flow nasal cannula therapy versus CPAP via different masks. Eur Respir J 2019;53 10.1183/13993003.02339-2018. 30705129

[23] Simonds AK, Hanak A, Chatwin M, Morrell MJ, Hall A, Parker KH, et al Evaluation of droplet dispersion during non-invasive ventilation, oxygen therapy, nebuliser treatment and chest physiotherapy in clinical practice: implications for management of pandemi. Health Technol Assess 2010;14:131–72. 10.3310/hta14460-02 20923611

[24] Frat JP, Ricard JD, Quenot JP, Pichon N, Demoule A, Forel JM, et al Non-invasive ventilation versus high-flow nasal cannula oxygen therapy with apnoeic oxygenation for preoxygenation before intubation of patients with acute hypoxaemic respiratory failure: a randomised, multicentre, open-label trial. Lancet Respir Med 2019;7:303–12. 10.1016/S2213-2600(19)30048-7 30898520

[25] Docherty AB, Harrison EM, Green CA, Hardwick HE, Pius R, Norman L, et al Features of 20 133 UK patients in hospital with covid-19 using the ISARIC WHO Clinical Characterisation Protocol: prospective observational cohort study. BMJ 2020;369:m1985 10.1136/bmj.m1985 32444460 PMC7243036

[26] Lawton T, Wilkinson KM, Corp A, Javid R, MacNally L, McCooe M, et al Reduced ICU demand with early CPAP and proning in COVID-19 at Bradford: a single centre cohort. MedRxiv 2020:202006.05.20123307. 10.1101/2020.06.05.20123307. PMC9679910 36751359

[27] Nightingale R, Nwosu N, Kutubudin F, Fletcher T, Lewis J, Frost F, et al Is continuous positive airway pressure (CPAP) a new standard of care for type 1 respiratory failure in COVID-19 patients? A retrospective observational study of a dedicated COVID-19 CPAP service. BMJ Open Respir Res 2020;7 10.1136/bmjresp-2020-000639. PMC7337881 32624495

[28] Oranger M, Gonzalez-Bermejo J, Dacosta-Noble P, Llontop C, Guerder A, Trosini-Desert V, et al Continuous positive airway pressure to avoid intubation in SARS-CoV-2 pneumonia: a two-period retrospective case-control study. Eur Respir J 2020:2001692 10.1183/13993003.01692-2020. PMC7241113 32430410

[29] Aliberti S, Radovanovic D, Billi F, Sotgiu G, Costanzo M, Pilocane T, et al Helmet CPAP treatment in patients with COVID-19 pneumonia: a multicenter, cohort study. Eur Respir J 2020:2001935 10.1183/13993003.01935-2020 32747395 PMC7397948

[30] Crimi C, Noto A, Cortegiani A, Impellizzeri P, Elliott M, Ambrosino N, et al Noninvasive respiratory support in acute hypoxemic respiratory failure associated with COVID-19 and other viral infections. Minerva Anestesiol 2020 10.23736/S0375-9393.20.14785-0. 32756535

[31] Schünemann HJ, Khabsa J, Solo K, Khamis AM, Brignardello-Petersen R, El-Harakeh A, et al Ventilation Techniques and Risk for Transmission of Coronavirus Disease, Including COVID-19: A Living Systematic Review of Multiple Streams of Evidence. Ann Intern Med 2020;173:204–16. 10.7326/M20-2306 32442035 PMC7281716

[32] Winck JC, Ambrosino N. COVID-19 pandemic and non invasive respiratory management: Every Goliath needs a David. An evidence based evaluation of problems. Pulmonology 2020;26:213–20. 10.1016/j.pulmoe.2020.04.013 32362507 PMC7183996

[33] He JL, Luo L, Luo ZD, Lyu JX, Ng MY, Shen XP, et al Diagnostic performance between CT and initial real-time RT-PCR for clinically suspected 2019 coronavirus disease (COVID-19) patients outside Wuhan, China. Respir Med 2020;168:105980 10.1016/j.rmed.2020.105980 32364959 PMC7172864

[34] Villarreal-Fernandez E, Patel R, Golamari R, Khalid M, DeWaters A, Haouzi P. A plea for avoiding systematic intubation in severely hypoxemic patients with COVID-19-associated respiratory failure. Crit Care 2020 241 2020;24:1–2. 10.1186/s13054-019-2683-3 32532318 PMC7291186

[35] Hui DS, Chow BK, Ng SS, Chu LCY, Hall SD, Gin T, et al Exhaled Air Dispersion Distances During Noninvasive Ventilation via Different Respironics Face Masks. Chest 2009;136:998–1005. 10.1378/chest.09-0434 19411297 PMC7094372

[36] Esquinas AM, Egbert Pravinkumar S, Scala R, Gay P, Soroksky A, Girault C, et al Noninvasive mechanical ventilation in high-risk pulmonary infections: A clinical review. Eur Respir Rev 2014;23:427–38. 10.1183/09059180.00009413 25445941 PMC9487394

[37] Radovanovic D, Rizzi M, Pini S, Saad M, Chiumello DA, Santus P. Helmet CPAP to Treat Acute Hypoxemic Respiratory Failure in Patients with COVID-19: A Management Strategy Proposal. J Clin Med 2020;9:1191 10.3390/jcm9041191. PMC7230457 32331217

[38] Mekontso Dessap A. Frugal innovation for critical care. Intensive Care Med 2019;45:252–4. 10.1007/s00134-018-5391-6 30284635

[39] Sartini C, Tresoldi M, Scarpellini P, Tettamanti A, Carcò F, Landoni G, et al Respiratory Parameters in Patients With COVID-19 After Using Noninvasive Ventilation in the Prone Position Outside the Intensive Care Unit. JAMA 2020. 10.1001/jama.2020.7861. PMC7229533 32412606

[40] Elharrar X, Trigui Y, Dols A-M, Touchon F, Martinez S, Prud’homme E, et al Use of Prone Positioning in Nonintubated Patients With COVID-19 and Hypoxemic Acute Respiratory Failure. JAMA 2020 10.1001/jama.2020.8255. PMC7229532 32412581

[41] Sarma A, Calfee CS. Prone Positioning in Awake, Nonintubated Patients With COVID-19. JAMA Intern Med 2020 10.1001/jamainternmed.2020.3027. 32584940

[42] Thompson AE, Ranard BL, Wei Y, Jelic S. Prone Positioning in Awake, Nonintubated Patients With COVID-19 Hypoxemic Respiratory Failure. JAMA Intern Med 2020 10.1001/jamainternmed.2020.3030. PMC7301298 32584946

[43] Telias I, Katira BH, Brochard L. Is the Prone Position Helpful During Spontaneous Breathing in Patients With COVID-19? JAMA 2020 10.1001/jama.2020.8539. 32412579

[44] Caputo ND, Strayer RJ, Levitan R. Early Self‐Proning in Awake, Non‐intubated Patients in the Emergency Department: A Single ED’s Experience During the COVID‐19 Pandemic. Acad Emerg Med 2020;27:375–8. 10.1111/acem.13994 32320506 PMC7264594

